# Multimodal deep learning for breast tumor classification: Integrating mammography and ultrasound for enhanced diagnostic accuracy

**DOI:** 10.1002/acm2.70464

**Published:** 2026-01-18

**Authors:** Yu Yan, Yichen Xu, Ge Fang, Xu He, Yifei Qian, Wenwen Zhu

**Affiliations:** ^1^ Department of Medical Equipment Jiangsu Province Hospital of Chinese Medicine Affiliated Hospital of Nanjing University of Chinese Medicine Nanjing Jiangsu China; ^2^ School of Artificial Intelligence and Information Technology Nanjing University of Chinese Medicine Nanjing Jiangsu China

**Keywords:** attention mechanisms, breast tumor classification, mammography, multimodal, ultrasound

## Abstract

**Background:**

Deep learning has advanced breast tumor prediction research, but traditional single‐modality models limit feature diversity and accuracy.

**Purpose:**

To develop and validate a multimodal deep learning approach that combines mammography and ultrasound imaging for improved breast tumor classification and enhanced clinical decision‐making.

**Methods:**

This retrospective study analyzed 663 female patients with breast lesions from 2018 to 2021, including 384 benign and 279 malignant cases. The two‐stage prediction model employed improved modality‐specific attention mechanisms: efficient channel attention (ECA‐Net) for ultrasound and convolutional block attention module (CBAM) for mammography. The fused features were input into a stacking ensemble module with logistic regression (LR), support vector machine (SVM), random forest (RF), and Extra‐Trees (ET) as base learners, and multilayer perceptron (MLP) neural network as meta‐learner. Data was divided into training (464), validation (133), and test (66) sets with a 7:2:1 ratio.

**Results:**

The proposed multimodal prediction model—mammography ultrasound (MPM‐MU) achieved superior performance with an area under the receiver operating characteristic (ROC) Curve (AUC) of 87.9 ± 0.21%, representing improvements of 13.4% and 15.6% over attention‐enhanced mammography (74.5%) and ultrasound (72.3%) models, respectively. Ablation studies confirmed the effectiveness of both multimodal feature fusion and attention mechanisms in enhancing diagnostic performance.

**Conclusions:**

The multimodal prediction model—mammography ultrasound (MPM‐MU) with modality‐specific attention mechanisms demonstrated superior performance in distinguishing between benign and malignant breast tumors compared to single‐modality approaches. This approach assists radiologists in improving breast lesion classification accuracy and enhancing clinical decision‐making, potentially reducing unnecessary biopsies and improving diagnostic consistency.

## 1 INTRODUCTION

Breast cancer is the most common malignancy among women, ranking first in incidence among female malignant tumors, and has emerged as a global public health concern.^1^, ^2^ In 2022, approximately 20 million new cancer cases were diagnosed worldwide, with breast cancer accounting for approximately 11.6% of these cases.^3^ The incidence is increasing at an annual rate of 3%–4%.^4^ By 2050, global incidence is projected to rise by 38%, with mortality increasing by 68%.^5^, ^6^ Accurate differentiation between benign and malignant tumors is crucial for early detection, as timely treatment greatly improves patient survival and quality of life, while misdiagnosis delays therapeutic intervention.^7^, ^8^, ^9^ Radiological examinations, including mammography (MG) and ultrasonography (US), enable detection and differentiation of tumor characteristics such as masses, calcifications, architectural distortions, and asymmetric densities.^10^, ^11^ However, single‐modality imaging has inherent limitations.^12^ Mammography shows reduced sensitivity in dense breast tissue, while ultrasonography has limited resolution for distinguishing microcalcifications. Accurate prediction models play a crucial role in addressing these limitations and supporting clinical decision‐making.^13^ These models effectively differentiate between benign and malignant breast lesions, assisting clinicians in formulating more appropriate treatment strategies, thereby improving therapeutic outcomes and reducing misdiagnosis rates.

With advances in deep learning for mammography and ultrasonography, numerous studies have investigated differentiating benign and malignant breast tumors.^14^, ^15^, ^16^ Cao et al. (2017) evaluated various convolutional neural network (CNN)‐based approaches for tumor detection in breast ultrasound imaging.^17^ Becker et al. (2018) demonstrated that deep learning achieved diagnostic accuracy comparable to experienced radiologists with potential for real‐time analysis.^18^ Attention mechanisms have gained widespread application in breast cancer classification by effectively identifying and emphasizing critical image regions. Meng et al. (2022) proposed a Dual Global Attention Neural Network (DGANet) integrating bilateral spatial and global channel attention, improving average precision by 0.2%0‐5.9%.^19^ Xu et al. (2023) introduced a Regional Attention Multi‐Task Learning Network (RMTL‐Net), enhancing diagnostic efficiency by simultaneously performing tumor segmentation and classification.^20^ Beyond innovations in model architectures and attention mechanisms, data quality and quantity remain critical challenges. Cai et al. proposed an ultrasound augmentation method based on conditional generative adversarial networks (cGAN), applying image‐to‐image translation to generate synthetic images from segmentation masks, improving classification performance by approximately 12.85%.^21^ In multimodal research, Chunhapran and Yampaka (2021) integrated ultrasound and mammography using MobileNet architecture, demonstrating that multimodal approaches enhanced classification performance and offered perspectives for reducing unnecessary biopsies.^22^ Atrey et al. proposed a dual‐modal model combining CNN and Long Short‐Term Memory (LSTM), analyzing 43 paired images from 31 patients. By fusing mammographic and sequential ultrasound features, they enhanced classification accuracy and model generalization.^23^ Du et al. developed a multimodal model based on mammography and multi‐sequence magnetic resonance imaging (MRI), analyzing five imaging modalities from 132 patients (87 malignant, 45 benign). Ensemble learning integration outperformed single‐modality approaches.^24^ Unlike the small‐scale multimodal datasets commonly employed in existing research, this study utilized 663 paired dual‐modal cases, providing broader pathological coverage from common fibroadenomas to rare cystic adenocarcinomas. By using clinically routine mammography and ultrasound images, this approach maintains diagnostic performance while reducing examination costs, achieving a favorable balance between clinical applicability and cost‐effectiveness.

The contributions of the paper are as follows:

1. A novel modality‐specific attention mechanism framework is proposed for multimodal breast imaging. The framework incorporates efficient channel attention network (ECA‐Net) for ultrasound and convolutional block attention module (CBAM) for mammography. This design leverages the distinct imaging characteristics of each modality, substantially improving feature extraction capability and model robustness.
2. We construct a multi‐level classification architecture based on stacking ensemble strategies. The extracted multimodal features are fed into multiple base learners including logistic regression (LR), support vector machine (SVM), random forest (RF), and extra‐trees (ET). A multilayer perceptron (MLP) serves as the meta‐learner for final prediction optimization, significantly enhancing classification stability and accuracy.
3. We validate our method on a large‐scale clinical dataset comprising 663 pairs of dual‐modality cases collected from Jiangsu Province Hospital of Traditional Chinese Medicine during 2018–2021. The dataset covers various pathological types ranging from common fibroadenomas to rare cystic adenocarcinomas. Our multimodal prediction model achieves an AUC of 87.9%, accuracy of 80.5%, and specificity of 79.3%, outperforming existing single‐modal and multimodal fusion methods.

## 2 MATERIAL AND METHODS

### 2.1 Patient selection and criteria

**FIGURE 1:**
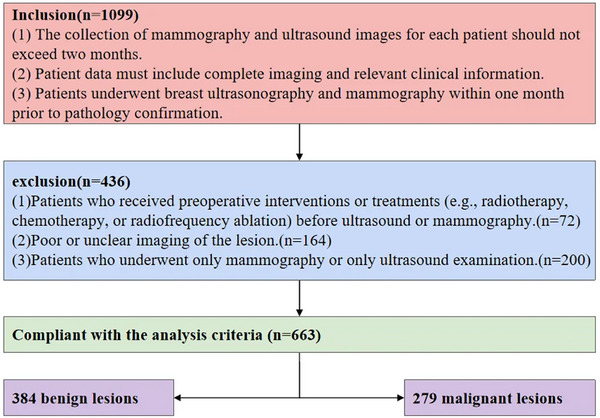
Flowchart showing study population and exclusion criteria.

This retrospective study was conducted in accordance with the Declaration of Helsinki and was approved by the Institutional Ethics Review Board. The requirement for informed consent was waived by the ethics committee. We analyzed 1,099 female patients with breast lesions treated at Jiangsu Province Hospital of Traditional Chinese Medicine from 2018 to 2021. The inclusion criteria for patients were as follows: (1) The collection of mammography and ultrasound images for each patient should not exceed 2 months. (2) Patient data must include complete imaging and relevant clinical information. (3) Patients underwent breast ultrasonography and mammography within 1 month prior to pathology confirmation. The exclusion criteria were as follows: (1) Patients who received preoperative interventions or treatments (e.g., radiotherapy, chemotherapy, or radiofrequency ablation) before ultrasound or mammography. (2) Poor or unclear imaging of the lesion. (3) Patients who underwent only mammography or only ultrasound examination. The flowchart of the study population and exclusion criteria is presented in Figure 1.

### 2.2 Image acquisition and equipment

Mammographic images were acquired using an IMS Giotto IMAGE 3D mammography scanner. For each patient, standard craniocaudal (CC) and mediolateral oblique (MLO) views were obtained for each breast following standard mammographic protocols. Ultrasound images were acquired using various US systems: 1. Philips iU‐22; 2. Philips EPIQ 7; 3. Mindray DC‐7; 4. GE LOGIQ S6; 5. Toshiba SSA‐790. All examinations were performed using high‐frequency linear transducers following standard breast ultrasound protocols.

### 2.3 Data preprocessing and augmentation

In the data preprocessing phase, we processed MG and US images from the same patient as paired inputs without explicit spatial registration between the two modalities. Each pair includes mammography images (CC and MLO views) and the corresponding ultrasound image. The model treats these images as parallel inputs, mimicking the clinical workflow of multimodal lesion assessment.

We first used an image annotation tool to crop the breast regions containing tumors from the original images.^25^ These regions were manually selected to include the tumor and surrounding breast tissue, and the presence of tumors within these cropped regions was confirmed by two radiologists with over 5 years of ultrasound interpretation experience. Our model took these cropped breast tissue regions as input.

**FIGURE 2:**
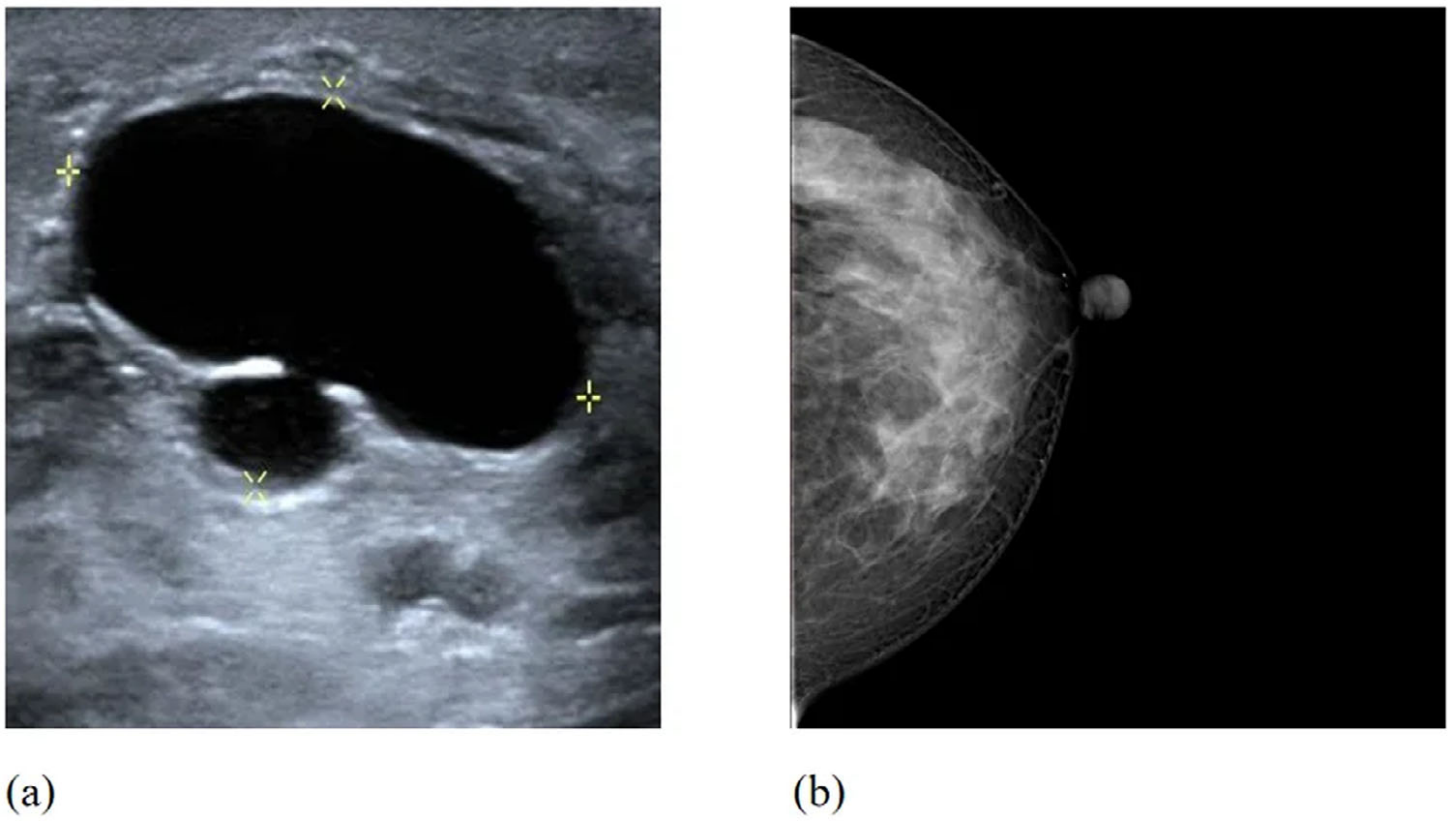
Cropped breast tissue regions used as model inputs: (a) ultrasound breast region containing tumor, (b) mammography breast region containing tumor.

For mammography images, preprocessing primarily involved cropping breast regions containing tumors and data augmentation. Since mammography images generally have high contrast and clear boundaries, breast tissue regions encompassing the tumor areas were manually selected and cropped from the original images. To improve the model's adaptability to images from different angles, augmentation of mammography images focused on rotation (±15°), affine transformations (degrees = 50), and flip operations (horizontal and vertical flipping, 50% probability each). In addition to spatial transformations, contrast adjustment (±50%) and brightness variation (±50%) were applied to account for variations between images. For ultrasound images, due to their less distinct boundaries compared to mammography images, the regions of interest (ROI) determination required radiologist annotation to ensure accurate bounding of the tumor area. Besides standard transformations including rotation (±10°), flipping, and affine transformations (degrees = 50), ultrasound images were further augmented with color distortion (including brightness ± 50%, contrast ± 50%, saturation ± 50%, and hue ± 50% adjustments) to improve the model's robustness to varying image characteristics across different imaging equipment and acquisition conditions.^26^ Finally, all cropped breast tissue regions were resized to a resolution of 256 × 256 pixels to ensure consistent input dimensions for the model. Figure 2a and b illustrates examples of the cropped breast tissue regions containing tumors from both mammography and ultrasound images, which served as the direct inputs to our classification model.

### 2.4 Model architecture

**FIGURE 3:**
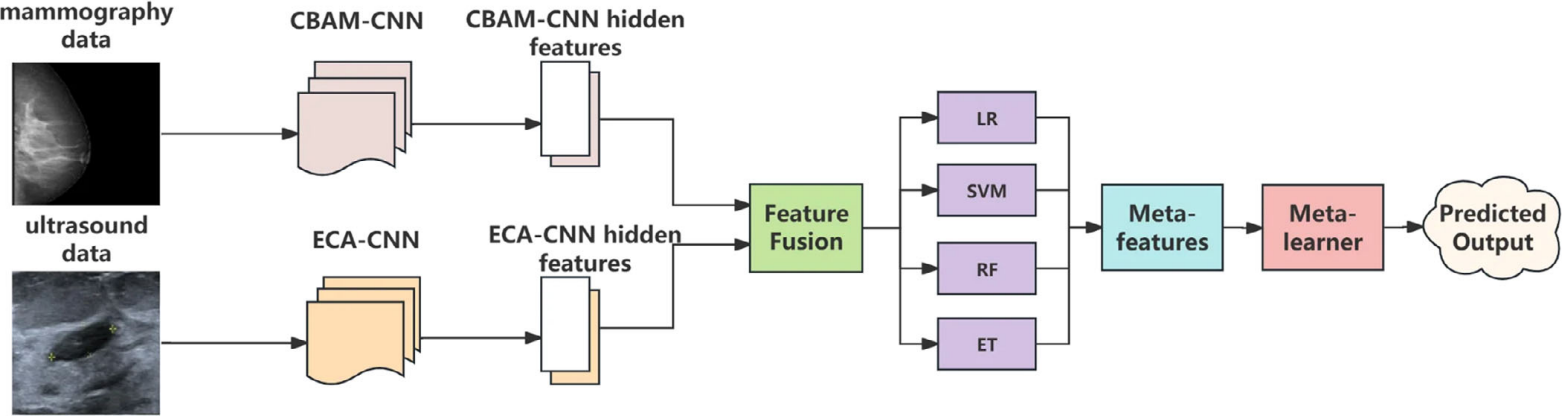
Flowchart of the multimodal prediction model—mammography ultrasound.

Given the limitations of single‐modality prediction, we developed a multimodal prediction model—mammography ultrasound (MPM‐MU), as illustrated in Figure 3. This model fully exploited the complementary advantages of mammography and ultrasound data for classifying patients with combined dual‐modality imaging to predict benign and malignant outcomes of breast tumors. Specifically, the model comprised two main components: The first component employed two specially optimized feature extraction branches, with the mammography branch incorporating CBAM and the ultrasound branch utilizing the ECA‐Net mechanism. Using ResNet‐18 as the feature extractor, hierarchical features were extracted from multiple layers of ResNet‐18 (Layers 1–4), followed by dimensionality reduction techniques for normalization and compression to retain over 95% of the original information. Finally, the 512‐dimensional feature vectors extracted from both branches are fused to form a 1024‐dimensional comprehensive feature representation. The second component fed these fused features into a stacking model that employs LR, SVM, RF, and ET as base learners. The predictions (positive class probabilities) from all base learners are combined to form enhanced meta‐features, including original probabilities, pairwise differences, pairwise products, and squared probabilities. These combined meta‐features are then passed to a single meta‐learner (an MLP neural network) to optimize the final prediction results and improve predictive performance.

**FIGURE 4:**
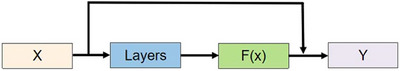
Residual connection.

Effective feature extraction is crucial for developing accurate prediction models. To establish an optimal feature extraction framework, we first conducted a comprehensive performance evaluation of five prominent CNN architectures: ResNet50, VGG16, AlexNet, Inception_V3, and ResNet18, considering both diagnostic performance and computational efficiency requirements for clinical applications. Based on this comparative analysis (detailed results presented in Section 3.2), ResNet18 was selected as it exhibited a favorable balance between performance (AUC 66.6%) and computational efficiency (9.53G FLOPS, 43.1MB). For mammography and ultrasound images, we employed a modified ResNet‐18 CNN to extract deep features. This architecture's effectiveness derived from its residual connections that alleviate the vanishing gradient problem, while its balanced performance‐efficiency ratio and relatively smaller parameter scale improve computational efficiency and reduce overfitting risk on our dataset.^27^ Each residual block in ResNet contained batch normalization layers, Rectified Linear Unit (ReLU) activation functions, and convolutional layers. To enhance the model's regularization capability and reduce overfitting risk, we introduced Dropout layers between convolutional layers and after each ReLU activation function.^28^ After the repetition of these operations, the results are added back to the input. This unique network structure, known as residual connections, enabled the input of each layer to be directly added to the output, making it particularly suitable for image classification tasks.^29^ An example of residual connections is illustrated in Figure 4, where X represents the input feature map, F(x) denotes the residual mapping, and Y represents the output feature map that is passed to the next residual block.

### 2.5 Attention mechanism design

The inherent differences in medical imaging modalities motivate the design of modality‐specific attention mechanisms. In ultrasound images, complex backgrounds and noise typically impede accurate feature extraction. To mitigate this challenge, attention mechanisms, inspired by the selective processing of human visual systems, adaptively enhance critical features through dynamic weight allocation. Although squeeze‐and‐excitation network (SE‐Net) and similar methods have been widely applied in medical imaging, their reliance on fully connected (FC) layers introduces computational burden and risks of feature degradation.^30^ ECA‐Net offers a lightweight solution to overcome these limitations by employing one‐dimensional convolution for local cross‐channel interactions while avoiding dimensionality reduction, thereby enabling feature response recalibration and noise suppression while preserving essential texture details.^31^, ^32^ In contrast, mammography images present distinct analytical challenges. Mammographic images typically exhibit high contrast with distinct structural boundaries and contain spatially localized diagnostic features such as microcalcifications, architectural distortions, and asymmetric densities. These features necessitate both channel‐wise feature refinement and spatial attention to precisely locate regions of interest.^33^ Islam et al. demonstrated that CBAM outperformed channel‐only attention mechanisms in mammographic breast lesion classification, effectively integrating spatial and channel attention to address the subtle and heterogeneous characteristics of breast lesions.^34^

**FIGURE 5:**
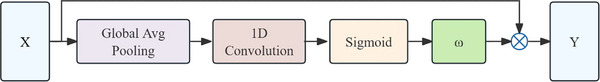
Network architecture of ECA‐Net.

For ultrasound images, as shown in Figure 5, the core mechanism of ECA involves capturing local cross‐channel dependencies through one‐dimensional convolution, with the channel weight calculation formula defined in Equation (1)^35^:

(1) $$\omega =\sigma \left(C1D\kappa \left(y\right)\right)$$

where y=g(X) represents the feature vector after global average pooling, X represents the feature map obtained after processing by the preceding convolutional layers. C1Dκ denotes one‐dimensional convolution with kernel size k, σ is the Sigmoid activation function, ω represents the channel weights, and the final output is Y=ω·X. The kernel size k was set to the default configuration and was determined adaptively based on the number of channels c, as defined by Equation (2). If k is an even number, then *k* = *k* + 1 to ensure k is always an odd value.

(2) $$k=\psi \left(C\right)=|\frac{\log_{2}\left(C\right)}{2}+\frac{1}{2}{|}_{odd}$$

**FIGURE 6:**
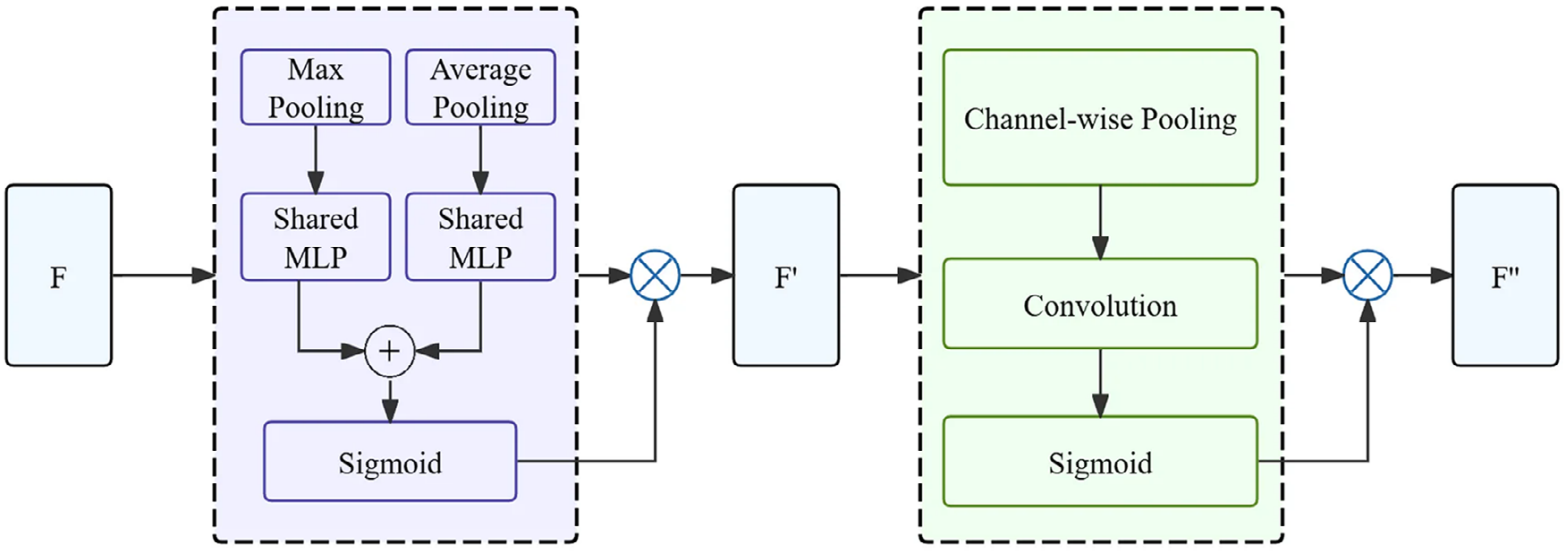
Network architecture of CBAM.

For mammography, Figure 6 illustrates the fundamental architecture of CBAM, which integrates channel and spatial attention sub‐modules to simultaneously enhance feature representation while preserving spatial information critical for detecting calcifications and structural abnormalities. The specific implementation steps, as defined in Equations (3) and (4), are as follows^36^:

The input feature map F undergoes both max pooling and average pooling to generate channel weights $M_{c}$, which are then multiplied with F:

(3) $$F^{\prime }=M_{c}\;\left(F\right)⊗F$$

$F^{\prime }$ is pooled along the channel dimension to generate a spatial attention map Ms, followed by another multiplication:

(4) $$F^{\prime \prime }=Ms\left(F^{\prime }\right)⊗F^{\prime }$$

where $M_{c}$ and Ms represent the channel attention and spatial attention sub‐modules respectively, and ⊗ denotes element‐wise multiplication.

### 2.6 Stacked model design

The stacking method enables the parallel utilization of multiple model predictions to generate a more optimized output. The core idea is to first use base learners to produce predictions (meta‐data) for the dataset, which are then processed by a meta‐learner. The base learners act as level‐0 learners, and the meta‐learner as the level‐1 learner, stacked on top of the base learners, hence the term “stacking”.^37^ In this model, the fused multimodal feature data was input into the stacking model. The input for the meta‐learner consisted of meta‐data generated by the base learners, and the classification results were produced through weighted integration. In the first layer of the module, we employed LR, SVM, RF, and ET as base learners, with the fused features represented as $\;X_{fused}$. The outputs of the base learners, given by Equations (5) and (6), are as follows:

(5) $$hLR=LR\left(X_{fused}\right),\;hSVM=SVM\left(X_{fused}\right)$$

(6) $$hRF=RF\left(X_{fused}\right),\;hET=ET\left(X_{fused}\right)$$

hLR, hSVM,hRF, and hET represent the prediction outputs of LR, SVM, RF, and ET, respectively. The outputs of the base learners were fed into the meta‐learner for final classification. An MLP served as the meta‐learner, producing the final classification output through non‐linear transformations of the base learners’ predictions. The prediction result of the meta‐learner is given by Equation (7):

(7) $$\begin{array}{cc} ypred & =f_{MLP}\left(w_{LR}\cdot hLR+w_{SVM}\cdot hSVM\right.\\ & \;+\left.w_{RF}\cdot hRF+w_{ET}\cdot hET+b\right) \end{array}$$

where $f_{MLP}$ represents the non‐linear transformation function of the MLP, $w_{LR}$, $w_{SVM}$, $w_{RF}$, and $w_{ET}$ are the weights assigned by the meta‐learner to the outputs of the base learners, and b is the bias term. The MLP applies multiple layer transformations using ReLU activation functions, with internal parameters optimizing weight distribution and bias terms added at each layer.

### 2.7 Multimodal training strategy

Given the distinct imaging characteristics of mammography and ultrasound modalities, we developed a differentiated training strategy that optimizes feature extraction performance for each modality while ensuring effective cross‐modal integration. For mammography images, CBAM was extensively applied across network layers to capture multi‐level spatial and channel features essential for detecting tissue density variations and mass margins. The configuration employed channel reduction ratio of 8, spatial attention kernel size of 3, and dropout rate of 0.3 applied between convolutional layers and after ReLU activations to enhance feature extraction capability. For ultrasound images, ECA‐Net was selectively configured in the deeper layers (layer3 and layer4) of ResNet18 to preserve channel feature integrity and enhance subtle texture differentiation. Systematic hyperparameter optimization was conducted for each modality branch. Both branches utilized AdamW optimizer with initial learning rates set to 5e‐5, beta1 = 0.9, and beta2 = 0.999, but with different weight decay values of 1e‐5 and 1e‐3 respectively. Learning rate scheduling employed ReduceLROnPlateau, with factors of 0.7 and patience of 8 for the mammography branch, and factors of 0.5 and patience of 3 for the ultrasound branch. Both branches were trained with batch size of 8 for 60 epochs.

For the base learners in the stacking module, Logistic Regression employed L2 regularization with regularization strength C = 1.0 and solver = “liblinear”. SVM utilized RBF kernel with gamma = “scale”. Random Forest was configured with n_estimators = 200 and min_samples_leaf = 4. Extra Trees implemented max_features = “sqrt” strategy. All base learners employed class_weight = “balanced” to address class imbalance issues. The meta‐learner utilized a three‐layer MLP with hidden layer sizes of (64,32,16) and ReLU activation functions, using Adam optimizer with learning rate of 0.001. Stratified random sampling was employed to partition the data into training (*n* = 464), validation (*n* = 133), and test (*n* = 66) sets at a 7:2:1 ratio based on benign/malignant labels.^38^ This ensured consistent class distribution across subsets. The test set remained completely independent throughout model development, including architecture selection and hyperparameter optimization. Model selection was based on the highest AUC on the validation set. The best checkpoint was saved for final evaluation. The test set was used exclusively for final performance assessment, following best practices to prevent data leakage. To evaluate the stability of MPM‐MU model performance, three independent runs were conducted. Results are reported as mean ± standard deviation.

The training and testing framework was implemented using PyTorch 2.1.1 with CUDA 11.8 support. Experiments were conducted on a system equipped with an Intel Core i7‐10700K CPU (3.8GHz, 8 cores), 32GB RAM, and an NVIDIA GeForce RTX 2080 Ti GPU (11GB VRAM).

## 3 RESULTS

### 3.1 Patient characteristics

This study collected data from 1099 female patients at Jiangsu Province Hospital of Traditional Chinese Medicine from 2018 to 2021, with 663 patients ultimately included in the analysis. The age range was 22–77 years, with a mean age of 49.2 years. There were 384 benign cases (mean age 49.4 years), including 210 cases of fibroadenoma, 35 cases of intraductal papilloma, and 139 cases of adenosis and other benign lesions; and 279 malignant cases (mean age 49.5 years), mainly comprising 224 cases of invasive breast cancer, 48 cases of ductal carcinoma in situ (DCIS), and seven cases of other malignant tumors. Table 1 shows the basic information of the patient population.

**TABLE 1:** Summary of patient demographic information.

Characteristic	Total	Benign	Malignant
Patients (*n*)	663	384	279
Age			
Mean (y)	49.2 ± 12.5	49.4 ± 12.5	49.5 ± 12.4
Range (y)	22–77	22–74	30–77
Lesion type, *n* (%)			
Benign	384 (57.9%)	384 (100%)	NA
Fibroadenoma	210 (31.7%)	210 (54.7%)	NA
Intraductal papilloma	35 (5.3%)	35 (9.1%)	NA
Adenosis and other benign lesions	139 (21.0%)	139 (36.2%)	NA
Malignant	279 (42.1%)	NA	279 (100%)
Invasive breast cancer	224 (33.8%)	NA	224 (80.3%)
Ductal carcinoma in situ (DCIS)	48 (7.2%)	NA	48 (17.2%)
Other malignant tumors^a^	7 (1.1%)	NA	7 (2.5%)

^a^ Including lobular carcinoma in situ, encapsulated papillary carcinoma, adenoid cystic carcinoma, and mucinous carcinoma.

### 3.2 Comparative analysis of different convolutional neural network architectures

As described in Section 2.4, we conducted a comprehensive performance evaluation of five prominent CNN architectures to establish the optimal feature extraction framework. The evaluation results, as presented in Table 2, demonstrate that VGG16 achieved the highest accuracy (ACC) of 68.4% and high specificity (83.2%), while ResNet18 exhibited a favorable balance between performance (AUC 66.6%) and computational efficiency (9.53G FLOPS, 43.14MB). Given the imperative balance between diagnostic performance and computational efficiency in clinical applications, ResNet18 was ultimately selected as the foundational architecture for subsequent experimentation.

**TABLE 2:** Performance and computational complexity comparison of different CNN architectures.

Model	AUC (%)	ACC (%)	Sensitivity (%)	Specificity (%)	F1‐score (%)	FLOP(G)	Model size (MB)
ResNet50	59.2	61.0	49.0	69.7	50.7	21.6	93.7
VGG16	65.4	68.4	48.1	83.2	55.7	40.3	512
AlexNet	56.4	62.0	24.1	90.3	33.1	3.69	217
Inception_V3	61.3	60.1	68.4	54.1	58.9	15.5	87.1
ResNet18	66.6	63.9	40.8	80.6	50.6	9.53	43.1

### 3.3 Comparative analysis of attention mechanisms across different medical imaging modalities

To evaluate the efficacy of attention mechanisms in multimodal diagnostic frameworks, we systematically investigated the performance of ECA‐Net and CBAM attention mechanisms on both mammography and ultrasound modalities. As illustrated in Table 3, the implementation of CBAM attention mechanism in mammographic imaging (ResCBAM) demonstrated superior performance, achieving an AUC of 74.5%, outperforming the baseline model. Conversely, in ultrasound imaging, the ECA‐Net attention mechanism (ResECA) exhibited optimal performance with an AUC of 72.3%.

**TABLE 3:** Performance comparison of attention mechanisms across imaging modalities.

Modality	Method	AUC (%)	ACC (%)	Sensitivity (%)	Specificity (%)	Precision (%)	F1‐score (%)
Mammography	ResNet18	66.6	63.9	40.8	80.6	61.8	50.6
	ResECA	64.4	59.7	96.2	33.0	48.3	63.2
	ResCBAM	74.5	62.9	65.1	62.6	53.5	60.7
Ultrasonography	ResNet18	65.5	67.6	51.8	79.2	64.4	57.4
	ResCBAM	71.9	57.1	92.9	32.5	49.5	64.1
	ResECA	72.3	69.1	67.8	70.1	62.3	64.9

### 3.4 Multimodal prediction model—mammography ultrasound performance

**FIGURE 7:**
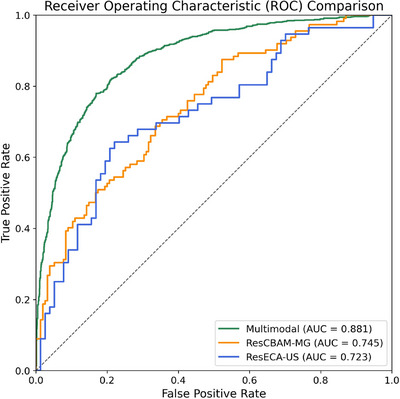
ROC curve comparison between single‐modality and multimodal models.

To validate the performance and stability of the MPM‐MU model, we conducted multiple independent runs and comparative experiments against single‐modality models enhanced with attention mechanisms. The three independent runs yielded AUC values of 87.8%, 88.1%, and 87.7%, with a mean AUC of 87.9 ± 0.21% and a mean accuracy of 80.5 ± 0.35%. The small standard deviations (0.21‐0.38% across metrics) demonstrated the robustness and reproducibility of the model training process. As shown in Table 4, the integrated multimodal approach demonstrated superior diagnostic capabilities across multiple evaluation metrics. The MPM‐MU model achieved a mean AUC of 87.9%, substantially outperforming other models and exhibiting enhanced predictive capability. In comparison, ResCBAM‐MG and ResECA‐US achieved AUC values of 74.5% and 72.3% respectively, showing a notable difference. This improvement in discriminative ability confirmed the effectiveness of our fusion strategy in integrating complementary information from different imaging modalities, thereby mitigating the inherent limitations and potential errors associated with single‐modality approaches. Figure 7 presents the comparative ROC (Receiver Operating Characteristic) analysis of the three different models. The MPM‐MU curve shown corresponds to the best‐performing run.

**TABLE 4:** Performance of multimodal prediction model—mammography ultrasound.

Model	AUC (%)	ACC (%)	Sensitivity (%)	Specificity (%)	Precision (%)	F1‐score (%)
ResCBAM‐MG	74.5	62.9	65.1	62.6	53.5	60.7
ResECA‐US	72.3	69.1	67.8	70.1	62.3	64.9
MPM‐MU	87.9 ± 0.21	80.5 ± 0.35	82.0 ± 0.38	79.3 ± 0.32	73.9 ± 0.30	77.5 ± 0.33

### 3.5 Ablation study on feature fusion and attention mechanisms

To systematically quantify the contributions of feature fusion and attention mechanisms in the MPM‐MU model, two independent ablation experiments were conducted. The first study examined the effectiveness of multimodal feature fusion, while the second evaluated the impact of attention mechanisms on diagnostic performance.

In the first ablation experiment, we investigated the impact of removing the feature fusion step. Features were extracted from ultrasound and mammography modalities and then separately input into the stacking module for training. The results of this configuration were compared with the performance of the MPM‐MU model. The ablation results demonstrate that multimodal feature fusion substantially improved performance, with the complete model achieving an AUC of 87.9% and accuracy of 80.5%, outperforming single‐modality approaches without feature fusion (Table 5).

**TABLE 5:** Ablation study results.

Configuration	Model	AUC (%)	ACC (%)	Sensitivity (%)	Specificity (%)	Precision (%)	F1‐score(%)
Single‐modal	Stacked‐MG	74.3	67.4	55.4	76.1	62.6	58.7
	Stacked‐US	64.8	65.4	50.0	76.6	60.9	54.9
Multimodal	No attention	68.9	65.2	34.5	87.5	66.7	45.5
	MPM‐MU	87.9	80.5	82.0	79.3	73.9	77.5

The second ablation experiment evaluated the contribution of attention mechanisms by comparing the complete model with a model without attention mechanisms (No Attention). Compared to the complete MPM‐MU model (AUC: 87.9%, Accuracy: 80.5%), the model without attention mechanisms achieved only 68.9% AUC and 65.2% accuracy. This represents a decline of 19.0 percentage points in AUC and 15.3 percentage points in accuracy, underscoring the critical contribution of attention mechanisms to diagnostic capability. Notably, the “No Attention” model exhibited unexpectedly high specificity (87.5%) but markedly low sensitivity, yielding an F1‐score of only 45.5%. This trade‐off indicates that the “No Attention” model adopts an overly conservative prediction strategy, reducing false positives at the expense of increased false negatives. This pattern highlights the essential role of attention mechanisms in capturing malignant features while maintaining balanced diagnostic performance.

### 3.6 Classification performance evaluation

The evaluation metrics for breast tumor classification predicted by different models are presented in Table 6. The MPM‐MU model achieved an AUC of 87.9%, representing improvements of 30.1%, 20.4%, 13.1%, and 10.3% over LR, SVM, RF, and ET, respectively. The consistent enhancements across all performance metrics underscore the robustness and reliability of the MPM‐MU model in multimodal breast tumor classification. These findings suggest that integrating features from multiple modalities through stacking methods offers substantial clinical value, with promising potential to improve diagnostic accuracy in breast cancer screening and diagnostic applications.

**TABLE 6:** Comparative analysis of classification methods for multimodal breast tumor prediction *p* < 0.01*.

Method	AUC (%)	ACC (%)	Sensitivity (%)	Specificity (%)	Precision (%)	F1‐score (%)
LR	57.8	59.3	32.3	78.9	50.7	38.6
SVM	67.5	65.7	52.1	75.6	58.9	53.4
RF	74.8	70.3	65.6	73.7	62.9	63.2
ET	77.6	72.9	66.2	77.8	66.9	66.4
MPM‐MU	87.9	80.5	82.0	79.3	73.9	77.5

*Statistical significance of AUC differences was assessed using DeLong's test.

## 4 DISCUSSION

This study investigated the application of deep learning‐based multimodal approaches in predicting the benign or malignant nature of breast tumors. We constructed a dual‐modal clinical dataset incorporating mammography and ultrasound images. By integrating features from different imaging modalities through a CNN architecture and machine learning algorithms, we achieved effective prediction of breast tumors.

Although mammography is the gold standard for screening, its sensitivity is greatly reduced in dense breasts. Furthermore, ultrasound diagnosis is prone to false‐positive issues. These limitations of single‐modality imaging stem from factors such as tissue overlap, density variations, and diagnostic subjectivity, often leading to diagnostic inconsistencies.^39^, ^40^, ^41^, ^42^, ^43^ Therefore, the American College of Radiology (ACR) and European Society of Breast Imaging (EUSOBI) guidelines recommend multimodal assessment for specific populations.^44^, ^45^ This study addressed this need by integrating multimodal information through deep learning techniques to enhance diagnostic accuracy and consistency, thereby supporting clinical decision‐making and reducing unnecessary biopsies.

Experimental results demonstrated that attention mechanism effectiveness varies across imaging modalities. As shown in Table 3, in mammography, ResCBAM achieved the highest AUC (74.5%). This indicated that its joint spatial‐channel attention effectively captured critical diagnostic features such as microcalcifications. However, in ultrasound imaging, although ResCBAM exhibited high sensitivity (92.9%), its accuracy (57.1%) and specificity (32.5%) substantially declined. This can be attributed to two factors. First, ultrasound images contain complex spatial patterns (e.g., acoustic artifacts) with considerable noise. CBAM's spatial attention overfocused on these non‐specific patterns, increasing false positives. Second, CBAM's combined spatial‐channel attention introduces more parameters, making it prone to overfitting on high‐noise US data with limited training samples. In contrast, ResECA showed more balanced performance on ultrasound (AUC 72.3%, specificity 70.1%). This demonstrated that lightweight channel attention is more suitable for the ultrasound modality. These results revealed inherent limitations of single‐modality approaches: different attention mechanisms perform differently across modalities. Complex architectures do not necessarily bring performance gains and may instead impair generalization due to overfitting.

Multimodal fusion was therefore employed to address these limitations by generating a cross‐modal complementary effect. This effect originates from each modality's intrinsic strengths. MG demonstrates high sensitivity for microcalcifications, detecting lesions smaller than 0.5 mm. In contrast, US provides superior soft‐tissue contrast for characterizing masses and margins, particularly in dense breast tissue. The synergistic integration of these features enables a more comprehensive understanding of tumor characteristics than either modality alone. Consequently, the fusion process acts as an implicit regularization. It mitigates the overfitting and modality‐specific limitations observed in single‐modality approaches, enabling more precise feature identification.

To demonstrate the rationale of the stacking architecture, we conducted comparative experiments against simple MLP fusion. Results showed that stacking ensemble substantially outperformed simple fusion (AUC: 87.9% vs. 74.7%, an improvement of 13.2 percentage points; Accuracy: 80.5% vs. 66.1%, an improvement of 14.4 percentage points). This performance gain justifies the increased architectural complexity for medical diagnosis. Our meta‐feature design included probability outputs from base classifiers along with their pairwise differences and products. This aimed to capture complementary information between models. The strategy follows validated ensemble learning theory.^46^, ^47^ Although the stacking architecture is more complex, its modular design facilitates future optimization. As a clinical decision support tool, its computational cost remains within acceptable limits.

Furthermore, we conducted comparative validation against existing single‐modal and multimodal methods (Table 7). We reimplemented the method proposed by Zhang et al. and evaluated it on our dataset under identical experimental conditions, where our MPM‐MU model outperformed it across key metrics (AUC = 87.9%).^48^ Additionally, our results surpassed the performance benchmarks as originally reported by Li et al. and Falconi et al., confirming the superiority of our modality‐specific attention mechanism and stacked ensemble learning strategy.^49^, ^50^

**TABLE 7:** Performance comparison with other related studies.

Author	AUC (%)	ACC (%)	Sensitivity (%)	Specificity (%)	Precision (%)	F1‐score (%)
Ours	87.9	80.5	82.0	79.3	73.9	77.5
Zhang et al.	81.2	78.0	78.5	75.2	71.3	74.1
Li et al.	85.0	NA	NA	NA	NA	NA
Falconi et al.	84.4	NA	NA	NA	NA	NA

Abbreviation: NA, complete metrics not reported.

Beyond comparison with other deep learning methods, we benchmarked our model performance against radiologist diagnostics based on the observer study by Xu et al.^51^ In that study, radiologists using single modalities achieved the following performance: US (AUC 67.4%–73.7%, specificity 52.1%–64.6%, sensitivity 82.8%) and MG (AUC 73.3%–77.2%, specificity 60.4%–75.0%, sensitivity 79.3%–86.2%). In contrast, our proposed MPM‐MU model achieved an AUC of 87.9%, sensitivity of 82.0%, and specificity of 79.3%. While maintaining high sensitivity, the model improved both specificity and overall discriminative ability. These results suggest that deep learning models integrating multimodal information can surpass radiologists' performance in interpreting single modalities. This further supports the potential value of incorporating such models into screening workflows to augment radiologist performance.

In clinical practice, the model's robust overall performance enables its integration into clinical workflows as an efficient “second reader”. Its objective, integrative predictions can provide critical decision support for radiologists, helping to reduce false positives and diagnostic uncertainty associated with single‐modality approaches, thereby lowering unnecessary biopsy rates. Technically, the model's approximate 2‐s per‐case prediction time demonstrates potential for real‐time clinical deployment. With further validation and optimization on multi‐center, prospective datasets, this approach is expected to become a valuable tool for improving diagnostic consistency and achieving personalized precision care, particularly in settings with uneven distribution of medical resources.

Despite the promising results, this study has several limitations. Its single‐center, retrospective design and the limited sample size may affect the generalizability of the findings. To balance computational resources, images were down sampled to 256 × 256 pixels, potentially affecting sensitivity to microcalcifications. The lack of public dataset comparison restricts broader benchmarking, and the framework is more complex than single‐modal approaches. To address these limitations, future work will incorporate multi‐center datasets and higher‐resolution inputs. We will also optimize model architecture through pruning, distillation, and alternative fusion strategies to enhance clinical deployment efficiency. Additionally, we plan to establish a continuous learning system where pathology‐confirmed errors are fed back to the model for ongoing improvement.^52^ Meanwhile, clinical translation of the model requires regulatory approval pathways. Future work will focus on attention visualization studies to enhance interpretability.

## 5 CONCLUSION

This study addressed the issue of insufficient feature extraction in previous research that relied on single‐modality data, proposing a deep learning‐based multimodal predictive model. This model utilized multimodal imaging data to predict breast cancer and distinguish between benign and malignant cases. By integrating features extracted from ultrasound and mammography data, this model employed deep learning combined with stacking ensemble techniques to consolidate multimodal features, the model achieved a more comprehensive feature representation, thereby improving the accuracy of breast cancer classification. Experimental results indicated that the proposed model MPM‐MU performed well across various evaluation metrics, showing potential clinical application value in the medical field, particularly in reducing unnecessary biopsies and treatments.

## AUTHOR CONTRIBUTIONS

Study conception and design: Yu Yan and Yichen Xu. Data collection: Yifei Qian and Ge Fang. Analysis and interpretation of results: Xu He. Manuscript preparation: Wenwen Zhu and Ge Fang. Manuscript review: All authors.

## CONFLICT OF INTEREST STATEMENT

The authors declare no competing interests.

## ETHICS STATEMENT

This retrospective study was conducted in accordance with the Declaration of Helsinki and was approved by the Ethics Committee of Jiangsu Province Hospital of Traditional Chinese Medicine (approval number: 2023NL‐071‐01).

## Data Availability

The data that support the findings of this study are available from the corresponding author upon reasonable request.
